# Corticosteroids in COVID-19: pros and cons

**DOI:** 10.3389/fmed.2023.1202504

**Published:** 2023-08-14

**Authors:** Aymen Bahsoun, Yeva Fakih, Rana Zareef, Fadi Bitar, Mariam Arabi

**Affiliations:** ^1^Faculty of Medicine, American University of Beirut Medical Center, Beirut, Lebanon; ^2^Pediatric Department, American University of Beirut Medical Center, Beirut, Lebanon; ^3^Pediatric Department, Division of Pediatric Cardiology, American University of Beirut Medical Center, Beirut, Lebanon

**Keywords:** corticosteroids, glucocorticoids, steroids, COVID-19, SARS-CoV-2, coronavirus

## Abstract

The wide and rapid spread of the COVID-19 pandemic has placed an unanticipated burden on the global healthcare sector. This necessitated a swift response from the international community to reach a solution. Efforts were made in parallel to develop preventative and therapeutic modalities. Since then, drug repurposing has blossomed as a potentially rapid resolution and has included various agents with anti-viral and anti-inflammatory properties. Corticosteroids, being potent anti-inflammatory agents, have been placed under extensive investigation. Various trials have recorded the beneficial outcome of corticosteroids in decreasing the mortality and morbidity of COVID-19. With the high pace of escalating events, the quality and study design of clinical trials are varied. Therefore, this study aims to explore the role of corticosteroids in COVID-19 disease. It inspects the molecular, pharmacologic, and clinical proof behind this theory.

## 1. Introduction

Since its emergence in Wuhan in December 2019, and its transition from an epidemic in China to a global pandemic, the novel SARS-CoV-2 virus has crippled healthcare systems worldwide (1). Its rapid spread has proven to be a global threat, with over 600 million cases and 6 million deaths reported internationally (2). The virus belongs to the *Coronaviridae* family, *Betacoronavirus* genus and is related to the previously observed middle east respiratory syndrome-related coronavirus (MERS-CoV), as well as severe acute respiratory syndrome coronavirus 1 (SARS-CoV-1) (3). Coronavirus, a zoonotic virus which primarily resides in bats, has mutated extensively over the past decade in order to be able to infect a human host (4, 5). Following its species boundary extension, and after successfully establishing an animal-to-human transmission, coronavirus presented with a wide symptomatology which ranges from completely asymptomatic (6), to a mild-moderate disease not requiring hospitalization (7). Nonetheless, some patients might experience a severe lower respiratory tract infection that may contribute to greater morbidity and mortality especially when it progresses into acute respiratory distress syndrome (ARDS), or acute lung injury among others (8). The severe symptomatology and mortality of COVID-19 patients has been greatly linked to the existence of various preceding comorbid conditions such as: hypertension, diabetes, chronic obstructive pulmonary disease (COPD), chronic kidney disease (CKD), cerebrovascular disease, cardiovascular disease, etc. (8). Other risk factors include but are not limited to age, socioeconomic status, ethnicity, male gender, diet, lifestyle, and the quality of healthcare provided (9). Consequently, the severity of the disease dictates the treatment options, and given the pandemic-related crises, effective treatment has become an urgent need.

While massive efforts have been channeled into the prevention of the disease, including multiple global vaccination campaigns and government-enforced social restrictions, treatment options have been limited. Currently, the standard of care includes maintaining adequate oxygen level, fluid resuscitation, and ventilation when necessary (10). In patients requiring oxygen supplementation, corticosteroids have been shown, in multiple trials, to improve outcomes such as mortality (11–13). On a pharmacological level, the mainstay of treatment for COVID-19 involves the use of already discovered agents such as the nonsteroidal anti-inflammatory drug aspirin (14). Additionally, antiretroviral protease inhibitors used to treat human immunodeficiency virus (HIV), ritonavir/lopinavir, were also examined in the setting of COVID-19 as combination agents with or without other drug families such as nirmatrelvir-ritonavir (15). Other options include anti-interleukin monoclonal antibodies such as tocilizumab and sarilumab (15), various antiviral agents such as ribavirin and favipiravir (15), and anti-inflammatory agents like corticosteroids, and interferons have also been repurposed in an attempt to combat the global burden of COVID-19 (10). Additionally, remdesivir was also explored in the context of COVID-19 (16). And other agents such as the antimalarial drug hydroxychloroquine displayed mixed results (17).

Due to time constraints imposed by the abrupt emergence of the disease, the utilization of existing treatments has been the most efficient strategy in combating the burden of the disease. This is owed greatly to the results of several randomized control trials (RCTs) demonstrating a benefit from the use of these agents, with a couple of notable mentions such as the randomized evaluation of COVID-19 therapy (RECOVERY) trial (4, 5, 18–20). It is a large multicenter trial involving 176 centers across the United Kingdom. It was conducted with the aim of identifying treatments that may improve outcomes for COVID-19 patients. They randomized 6,425 patients (2,401 dexamethasone arm versus 4,321 usual care arm) to receive dexamethasone versus usual care. Additionally, smaller studies have found mixed result, which will be discussed later on. In this review, we are going to focus on corticosteroids, synthetic derivates of the natural steroid cortisol. With the rapid irresistible spread of the pandemic, these astonishing agents were used to treat patients with COVID-19 and have demonstrated a marked decrease in mortality in severe cases (13). The working theory is that steroids counter the inflammatory response, hence they might prevent the progression to a late, immune-mediated stage of COVID-19 disease (20). Consequently, the aim of this paper is to portray the therapeutic benefit of several corticosteroids in treating COVID-19, as well as to touch upon some of their failures and adverse outcomes.

## 2. Methodology

Two databases including Pubmed and Ovid Medline were searched until May 23, 2023 using both the keywords and MeSH terms including: “COVID-19” OR “Coronavirus” OR “SARS-COV-2” and “Dexamethasone” OR “Methylprednisolone” OR “Steroids” OR “Corticosteroids.” Articles included were randomized control trials (RCTs) and retrospective observational studies discussing the use of glucocorticoid among other commonly studied agents (hydroxychloroquine, ritonavir, ribavirin, lopinavir, remdesivir, aspirin, tocilizumab) in patients with throat-swab or PCR confirmed COVID-19 in adults aged more than 18 years. Exclusion criteria were articles discussing the use of agents outside the scope of the review (vitamin D, sex steroids, convalescent plasma), and articles discussing the treatment specific complications of COVID-19 [anosmia, multisystem inflammatory syndrome in children (MIS-C)]. Ongoing and prospective trials were excluded from this review. There was no exclusion on the basis of language.

Two authors (AB and YF) screened the abstracts and titles of relevant articles identified through the search. Articles were selected based on their relevance and the inclusion/exclusion criteria mentioned above. Duplicates were removed, and disagreements were resolved by discussion or by consulting a third author (RZ). The authors screened the reference lists of relevant articles to further identify potential articles that could be included in the study.

## 3. Clinical uses of glucocorticoids

The medicinal history of corticosteroids goes back to 1930 when studies showed that adrenocortical samples extracted from animals have the potential to combat failure of the adrenal glands when administered to humans (21). Following that, in 1940, corticosteroids were split into two groups, one displaying the ability to retain bodily fluid and sodium, while the other showing an anti-inflammatory potential (21). Later, in 1948, and for the first time, cortisone was administered to a patient suffering from rheumatoid arthritis (21). The latter marked the beginning of the long and successful journey of corticosteroids in the field of medicine. In the years that followed, multiple synthetic steroids were marketed (21). And by 1960, their entire side effect profile was meticulously explained (21).

Since the initial description of their effects on immunity, metabolism, and cognition in 1949, glucocorticoids have been put into clinical use for a variety of conditions (22). Their clinical use has been enhanced through the chemical modification of naturally occurring steroids, such as cortisol, into a variety of synthetic glucocorticoids with most exerting a more potent effect than their original molecules (23–25). It took about 20 years from discovering the formation of cholesterol, to establishing an effective formulation of a topical steroid in 1952 (26).

The nonendocrine uses of corticosteroids, achieved at supraphysiologic doses, are numerous and span over almost every area in medicine through almost all routes (27). They are mostly used as agents to treat a variety of conditions that involve dysfunction of the immune system, which can be allergic, autoimmune, or inflammatory in nature. They are also used for their ability to amplify the catecholamine response in the body which is helpful in increasing vascular tone in the setting of hypotensive shock (28).

The endocrine benefits of corticosteroids can be therapeutic or diagnostic for the treatment of dysfunctional adrenal glands (29). Since excess production of corticosteroids can cause Cushing’s syndrome, whereas their deficiency can lead to Addison’s disease, they are equally useful in the diagnosis and treatment of the aforementioned phenomena, with various doses being used to rule out the different etiologies of Cushing’s syndrome (29). Additionally, a physiologic dose of glucocorticoids is essential for patients with primary adrenal insufficiency whereby the primary failure is in the adrenal glands, or secondary adrenal insufficiency which is a result of pituitary dysfunction, to maintain the physiologic effect of cortisol (30). This is particularly important to consider in the peri-operative period, whereby supraphysiologic doses are required to withstand intervention (29).

## 4. Pharmacology

Corticosteroids can be delivered via multiple routes (31). For example, systemic steroids can be widely used to treat multiple inflammatory, allergic and immune mediated disorders, yet dosing is extremely important to prevent one of their notorious side effects: the suppression of the hypothalamic-pituitary-adrenal axis (31). Alternatively, topical or inhaled corticosteroids can be used in instances where a local effect is desired, such as in the case of treating chronic obstructive pulmonary disorder (COPD), or asthma where the target organ is the lung (31). Furthermore, intranasal corticosteroids can be used in mediating allergic rhinitis of a moderate-to-severe form (31).

It is important to mention that despite the local concentration of inhaled and topical corticosteroids, the difference and uniqueness of their systemic bioavailability largely dictates their resultant systemic side effects (31). In terms of some pharmacokinetic properties of certain corticosteroids, it has been shown that the oral bioavailability is the smallest, whereas the pulmonary residence is the greatest for fluticasone propionate (32). Alternatively, flunisolide and budesonide showed the smallest pulmonary residence (32). Also, in terms of half-life, short-to-medium intervening corticosteroids such as prednisone and methyl-prednisone exhibit a half-life ranging from 8 to 36 h, while the long-acting ones such as dexamethasone and betamethasone display a 36 to 54 h long half-life (33). When it comes to potency of their anti-inflammatory activity, prednisolone showed a 5 times greater anti-inflammatory potency, while dexamethasone and betamethasone showed a 25 to 30 times increase as compared to the synthetic cortisol, hydrocortisone (33). This is attributed to their longer plasma half-life, which is a consequence of their synthetic alteration from the original molecule cortisol, which leads to different receptor binding and hence slower degradation of the steroid molecule by the liver (34).

In brief, the routes of administration and dosing are extremely important in preventing more serious side effects such as steroid-induced osteonecrosis, whereby most cases were observed when patients with preexisting comorbidities were given high dosages of systemic corticosteroids (35). On another note, the dosing and type of corticosteroid used are important to consider in the case of pregnancy (33). Some corticosteroids such as prednisone and prednisolone are preferred for treating maternal disorders since they do not cross the placenta readily (33). Whereas others such as betamethasone and dexamethasone show extensive transplacental crossing and are therefore indicated for the treatment of fetal diseases, especially when the risk of preterm birth is particularly high (33).

The two main routes studied for the treatment of COVID-19 have been inhalation and systemic administration. For patients suffering from COVID-19 and requiring oxygen supplementation, based on mixed results from large trials such as the RECOVERY and COVID STEROID 2 trial, the most commonly used systemic steroid has been dexamethasone (18, 36, 37). In resource-limited settings dexamethasone might not be available, hence it has been deemed reasonable to administer an equivalent corticosteroid. Such alternatives could be hydrocortisone, methylprednisolone, or prednisone (38). On the other hand, inhaled corticosteroids have not been studied as extensively, and further trials are required to establish their role in the management of early, mild-moderate COVID-19 disease.

**Figure 1:**
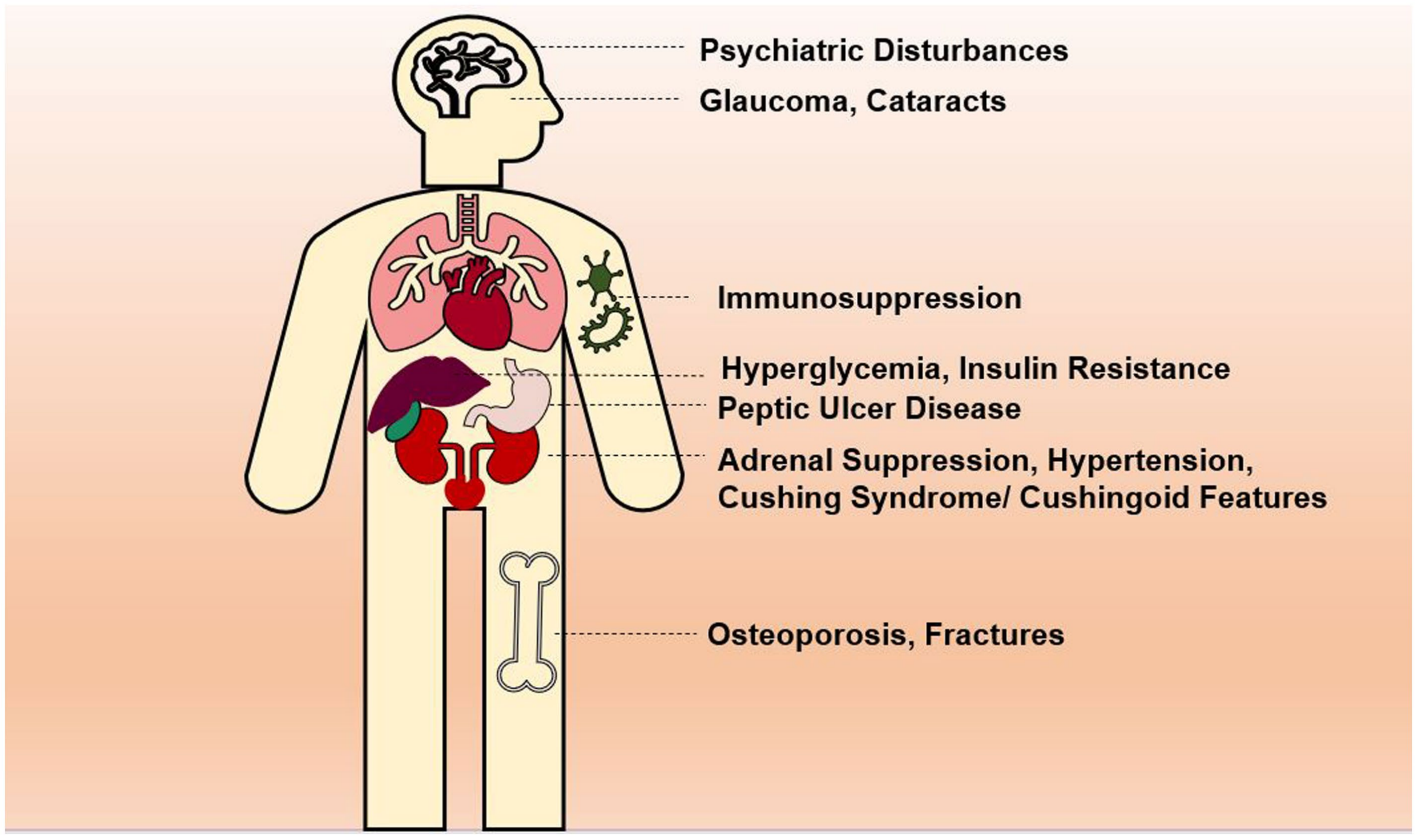
Side effects of chronic use of corticosteroids.

The most common side effects are usually associated with oral and systemic corticosteroids, and these include immunosuppression as patients face an increased risk for dangerous and uncommon infections (39). Additionally, since cortisol and corticosteroids bear an astonishing functional and structural similarity, the chronic intake of corticosteroids can potentially suppress the adrenal gland, leading to severe and potentially fatal adverse events such as hypotension (39). Also, iatrogenic corticosteroids are the primary cause for Cushing syndrome and the display of cushingoid characteristics (40). Although rare, some psychiatric side effects were also noted in a minority of patients, including psychosis (41, 42). The short use of corticosteroids is linked to less severe adverse events such as hyperglycemia, hypertension, electrolyte imbalance, and pancreatitis among others (41). Whereas the chronic use is related to more dangerous side effects like bone abnormalities and osteoporosis, hyperlipidemia, as well as negative outcomes on various organ systems such as the liver, eye, intestine and stomach (41), displayed in Figure 1. Unique to inhaled corticosteroids, patients can present with a fungal mouth infection (oral thrush/oral candidiasis) (43). On another note, antenatal corticosteroids have been linked to the dysfunction of the hypothalamic-pituitary-adrenocortical axis (44). In short, despite the wide range of adverse effects of corticosteroids on multiple organ systems, they can effectively be minimized when the course of treatment is short, and the dosages are progressively tapered down.

## 5. Mechanism of action

**Figure 2:**
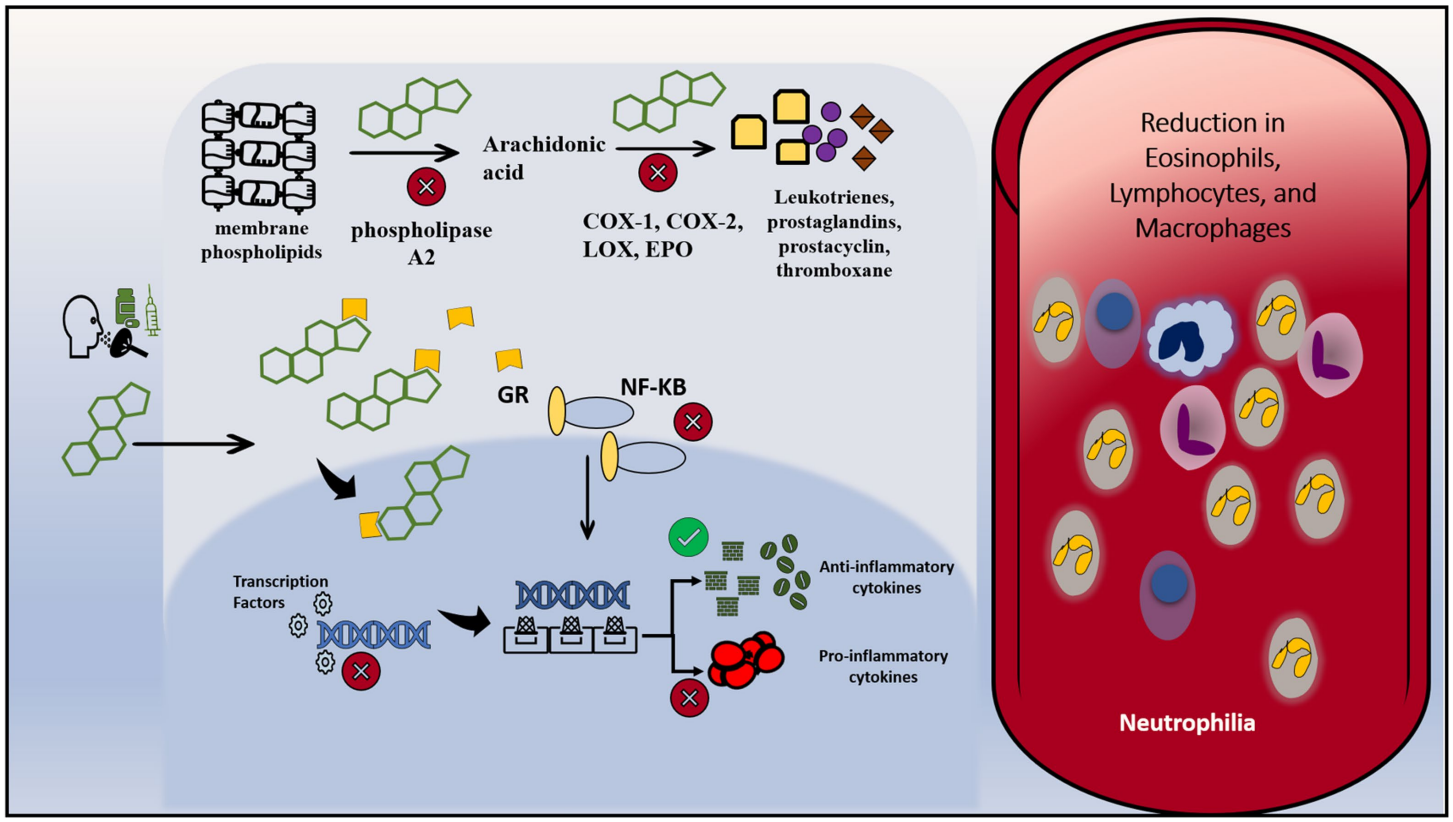
Mechanism of action of steroids. Corticosteroids can be administered orally, intravenously or via inhalation. Due to their lipophilicity, they can diffuse across the cell membrane and enter the cytoplasm. Inside the cell, they bind to intracytoplasmic glucocorticoids receptors, and combined cross to reach the nucleus. In the nucleus, they alter the transcription of multiple genes through intervening with the interaction between transcription factors and their target genes. They enhance the transcription of anti-inflammatory cytokines and inhibit the transcription of pro-inflammatory cytokines. On another level, they downregulate the activity of NF-KB, a prominent pro-inflammatory molecule. They bind to NF-KB and inhibit its signaling pathway and its subsequent interaction with the DNA. Besides, they interfere with the metabolism of membrane phospholipids by inhibiting vital enzymes: phospholipase A2, COX-1, COX-2, LOX and EPO. Therefore, the result is a decrease in vascular changes and cardinal inflammatory signaling. Intravascularly, it leads to a reduction in eosinophils, lymphocytes and macrophages and increase in intravascular neutrophils due to downregulation of leukocyte adhesion protein. COX-1, cyclooxygenase-1; COX-2, cyclooxygenase-2; LOX, lipoxygenase; EPO, peroxidase; GR, glucocorticoids receptor.

Corticosteroids, being structurally related to cortisol, are naturally lipophilic and are well renowned for their immunosuppressive effects (45). Given this lipophilicity, they passively diffuse across the cell membrane, bind to an intracellular glucocorticoid receptor, and migrate into the nucleus where they influence the transcription of multiple downstream genes. This is done by altering the affinity of transcription factors to promoter sites of said genes. The mechanism of action is depicted in Figure 2. First, they aim to selectively enhance the transcription of anti-inflammatory cytokines by the aforementioned mechanism (46). Second, they simultaneously block the promoter sites of genes that code for pro-inflammatory cytokines, as well as adhesion molecules that assist in inflammation (46). These include, but are not limited to, interleukins and eicosanoids such as interleukin(s) 1, 2, 6, tumor necrosis factor-alpha (TNF-a), granulocyte-macrophage colony-stimulating factor (GM-CSF), interferon-gamma (IFN-y) among others (47). Another major mediator of their immunosuppressive process is the downregulation of nuclear factor kB (Nf-kb), which plays a major role in mediating cellular signaling pathways that are involved in amplifying the immune response (46, 48).

Leukotrienes, prostaglandins, prostacyclins, and thromboxanes are among important pro-inflammatory cellular signaling molecules that are derived from membrane phospholipids of cells. Phospholipase A2 is an enzyme that works to initially catalyze the breakdown of membrane phospholipids into the aforementioned molecules. Not only have glucocorticoids been shown to inhibit phospholipase A2, but they furthermore inactivate the enzymatic conversion of downstream products by inhibiting enzymes such as cyclooxygenases 1 and 2 (COX-1, COX-2), lipoxygenases, and various synthases, similar to how non-steroidal anti-inflammatory drugs (NSAIDs) work, albeit in a more potent fashion (49). The result is a decrease in vascular changes and cardinal inflammatory signaling. Additionally, these agents also alter the physiology of cells that mediate inflammation in the body. This is done by changing basic immune cellular functions such as margination, migration, phagocytosis, and survival (50, 51). The effect on humoral immunity seems to be less profound than on cell-mediated immunity (38). Indeed, several lab studies corroborate these findings, which include the reduction of eosinophils, B, and more so T lymphocytes. There is a similar effect on macrophages, however, the effect is seen at much higher doses (52, 53). Neutrophils generally show an increase in number due to a decreased ability to adhere to endothelial walls (54). This is a consequence of the downregulation of leukocyte adhesion protein, resulting in an increase of neutrophils in the bloodstream from sites such as the bone marrow and vascular endothelia (54).

## 6. Corticosteroids and COVID-19

The rapid development of the COVID-19 pandemic necessitated the development of an efficient and effective response strategy. Treatment regimens that improve outcomes in severe infection were of particular interest. It seemed that the quickest way to achieve this was through utilizing medications that were already available for other diseases. Particular medications such as aspirin, specific immune modulators (e.g., anti-interleukin therapy), and corticosteroids were being studied (55, 56). Aspirin has been shown to be effective in the context of COVID-19 (14). As for anti-interleukin therapy, tocilizumab has been shown to be beneficial in hospitalized patients, especially when combined with corticosteroids, and sarilumab should only be used instead of tocilizumab when the latter is not available or there are contraindications against its usage (57–59). As for ritonavir and lopinavir, they were proven to be ineffective in the setting of COVID-19 (15, 17). Similarly, ribavirin demonstrated no benefit against COVID-19 (60). The use of favipiravir was likewise ineffective (15). A combination of nirmatrelvir-ritonavir (paxlovid) showed improved outcomes such as mortality (61). Remdesivir was the first antiviral agent approved for COVID-19 and has continued to be recommended as it has remained effective in improving outcomes in patients with mild to moderate COVID-19 (62, 63). Other antiviral agents approved for COVID-19 include molnupiravir and nirmatrelvir, and these agents have been showed to be continuously effective despite the emerging strains such as the Omicron variant (64). Also, evidence for agents such as the antimalarial drugs encompassing hydroxychloroquine and chloroquine have been shown to be ineffective in improving outcomes and potentially harmful (65).

**Figure 3:**
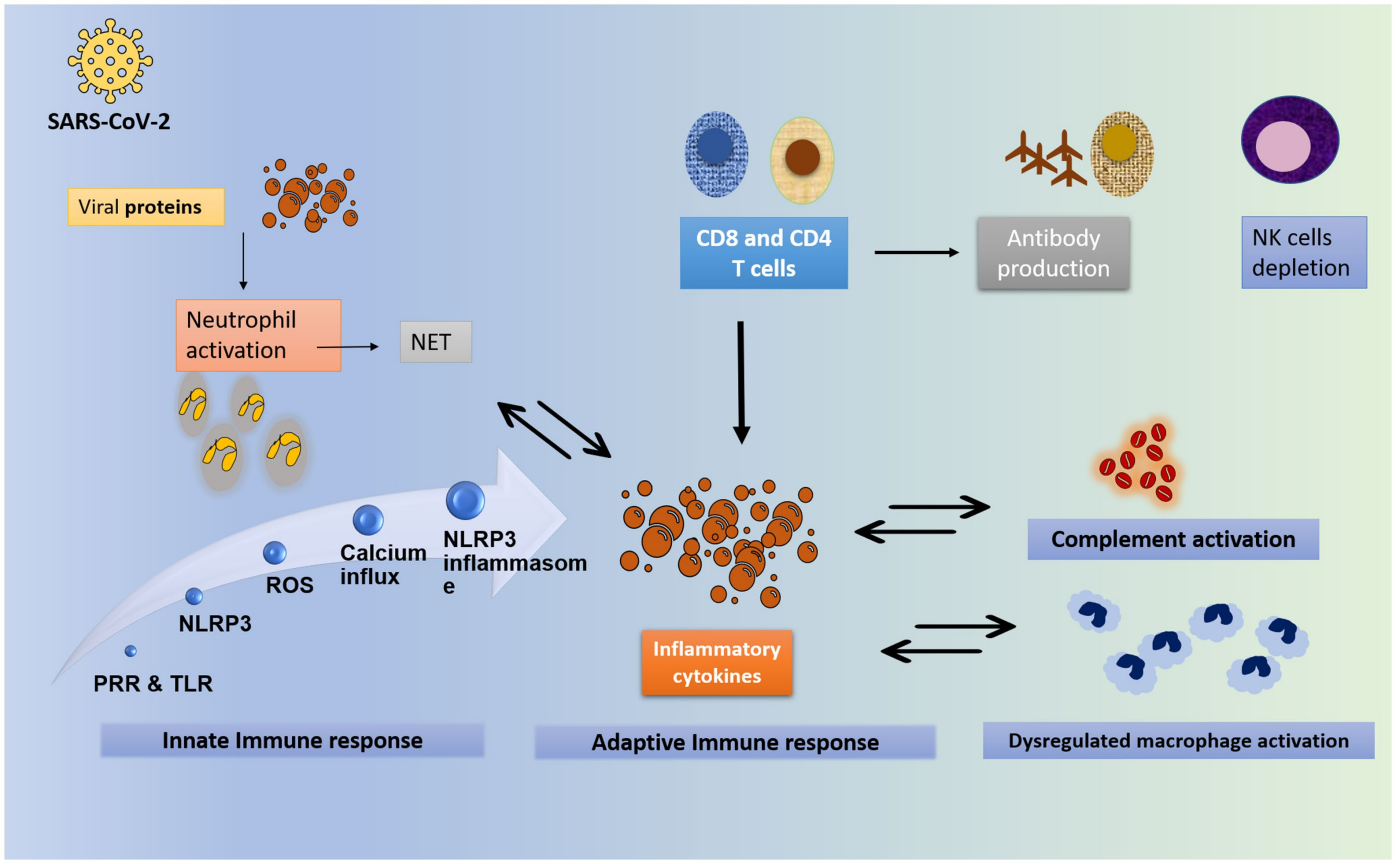
The hyperinflammatory state of COVID-19. The worsening and severe state of COVID-19 is attributed to the exaggerated inflammatory response rather than the viral load. The inflammatory process starts with the viral entry into the cell through binding to ACE2 receptors. Upon viral invasion of the target cell, the innate immunity is activated. Toll-like receptors and pattern-recognition receptor recognize the viral RNA and are activated. They in turn activate the NLR family pyrin domain containing 3 (NLRP3) gene. Similarly, the viral particles trigger the release of reactive oxygen species, protein aggregation and calcium influx. All these steps together constitute the NLRP3 inflammasome. At the same time, neutrophils are activated in response to the viral protein, to form neutrophil extracellular traps (NET), which further enhance the inflammatory response. In addition, NET formation is enhanced by inflammatory cytokines. Neutrophilia is usually noted. Part of innate response includes dysregulated activation of granulocytes and monocytes. The complement is an integral part of the innate immunity. It is activated directly by the viral proteins, or indirectly by immune complexes and TLR. It plays a central role in the development of hyperinflammatory state in COVID-19. Complement activation, especially C3 and C5A are suggested to contribute to ARDS formation. As for the adaptive immunity, T- and B- cells contribute to the response against viral invasion. T CD8- and CD4- lymphocytes are activated by viral proteins. Both T CD8- and CD4- release pro-inflammatory cytokines that enhance the cytokine storm, particularly IFN-gamma. CD8 cells activate B lymphocyte to release effective antibodies. Of note, the T cell count with its both arms CD4 and CD8 is shown to be significantly reduced in severe disease, with an increase in Th17 counts and activity (68–70). NET: neutrophils extracellular trap; PRP: pattern recognition receptors; TLR: toll like receptors; NRLP3: NLR family pyrin domain containing 3 gene; ROS: reactive oxygen species; NK: natural killer.

Corticosteroids have been shown *in vivo* to reduce inflammation that is associated with a dysregulated immune response, hence potentially reducing mortality if given early in the severe stage of the illness (66). In the context of a severe COVID-19 case, where the infection has not been cleared by the initial immune response and has entered the pulmonary phase, the proposed benefit of introducing corticosteroids is thought to be due to the downregulation of immune-mediated lung injury and cytokine storm (67). The hyperinflammatory state of COVID-19 disease is depicted in Figure 3. This would prevent the progression to acute respiratory distress syndrome (ARDS), respiratory failure, or death. Indeed, it has been suggested that SARS-CoV-2 induces the elevation of cytokines such as IL-6, potentially leading to a cytokine storm, an important contributor to mortality (67). This is supported by the finding of elevated levels of cytokines in critical COVID-19 patients, and these levels are directly correlated with higher mortality and a poorer prognosis (67).

An extremely significant disadvantage to the use of steroids in general is their important adverse effect profile. Systemic steroids can affect multiple organ-systems and lead to various side effects, and is generally related to the average dose and duration of treatment. These include: immunosuppression with predisposition to opportunistic infections, osteoporosis, osteonecrosis, fractures, hyperglycemia, promote insulin resistance, Cushing syndrome or cushingoid features, psychiatric disturbances, hypertension, adrenal suppression, glaucoma and cataracts. However, as these issues are dependent on the average dose of corticosteroids and the cumulative duration of treatment, the issue of adverse effects is minor in the short-term treatment of COVID-19 (27, 71–77).

### 6.1. Pros

There are many reasons that make corticosteroids a favorable candidate for the treatment of COVID-19. It is a drug that is used for numerous other conditions with a side effect profile that is well understood and justifiable when considering the risk-to-benefit ratio (78). Due to their widespread usage, they are relatively inexpensive and readily available. Recent bioinformatic and experimental evidence for immunomodulator use in severe COVID-19 infection has suggested that a pure anti-viral strategy is ineffective in later stages, as the viral load drastically drops (4, 19). However, the key practical limitation is the lack of large blinded randomized control trials for anti-viral agents (4, 19). A number of large trials have shifted the paradigm of the treatment of COVID-19 and recently published systematic reviews/meta-analyses have highlighted the widespread use of corticosteroids, most notably dexamethasone (59, 79–81), as highlighted in Table 1. These reviews have portrayed the potential of these agents to decrease mortality in hypoxemic patients patients requiring oxygen supplementation (59, 79–81). The same benefit has not been observed in patients with mild COVID-19 symptoms (59, 79–81). A notable example of such investigations is the RECOVERY trial (18). It is a large multi-center RCT that assigned patients to receive either 6 mg of dexamethasone, once daily, for 10 days utmost, following an intravenous (IV) or oral route of administration or usual care. This trial was the first and largest RCT published that provided evidence for mortality reduction (18). It included 2,104 participants receiving dexamethasone and 4,321 participants receiving usual care (18). This landmark trial was able to provide evidence for a decrease in the primary outcome, mortality at 28 days (29.3% treatment group vs. 41.4% standard care; rate ratio, 0.64; 95% CI: 0.51–0.81), when given 6 mg of dexamethasone for 10 days or until discharge. However, the greatest benefit that they observed was in hospitalized patients that has oxygen requirements or mechanical ventilation (MV), and the benefit was less in those requiring oxygen therapy without invasive MV (23.3% vs. 26.2%; rate ratio, 0.82; 95% CI: 0.72–0.94) (18). The CoDEX trial was another open label randomized control trial (RCT) that included 299 patients and found that at 28 days following the initiation of treatment there was a significant difference only in their primary outcome, ventilator-free days, which is defined as days alive and free of MV (37). The ventilator-free days were 6.6 days (95% CI: 5.0–8.2) in the dexamethasone plus standard care group versus 4.0 (95% CI: 2.9–5.4) in the standard care alone (37). It is important however to note that the study is limited by its open label design, as well as its early termination of enrollment following the release of the RECOVERY trial results (37). Additionally, about a third of participants in the standard care group had received corticosteroids (37).

**Table 1:** High impact studies that displayed efficacy of steroids in patients with COVID-19.

Study	Study type	Country	Study size	Agent used	Primary outcome
Dexamethasone in Hospitalized Patients with COVID-19 *The RECOVERY Collaborative Group* (18)	Multicenter, open label RCT	United Kingdom	6,425	Oral or IV dexamethasone (6 mg) for up to 10 days	Lower incidence of mortality at 28 days in the treatment group among participants receiving invasive mechanical ventilation (more pronounced) or oxygen supplementation without invasive mechanical ventilation
Effect of dexamethasone on days alive and ventilator-free in patients with moderate or severe acute respiratory distress syndrome and COVID-19. *The CoDEX Randomized Control trial* (37)	Multicenter, open label RCT	Brazil	299	20 mg dexamethasone for 5 days, followed by 10 mg for 5 days or until ICU discharge	A higher number of ventilator-free days during the first 28 days, defined as being alive and free from mechanical ventilation, in the treatment group
Intravenous methylprednisolone pulse as a treatment for hospitalised severe COVID-19 patients: results from a randomised controlled clinical trial (82)	Single-blind RCT	Iran	68	IV methylprednisolone pulse (250 mg) for 3 days	Clinical improvement was more significant and the mortality rate was noticeably lower in the treatment group
Open-label RCT of inhaled budesonide in nonhospitalized patients with COVID-19. *The PRINCIPLE trial* (83)	Multicenter, open-label RCT	United Kingdom	4,700	Inhaled budesonide	Decreased self-reported time to recovery and potentially reducing COVID-19 related hospital admissions and deaths

There is an important albeit unknown question of which dosage of dexamethasone might achieve the intended effect in improving outcomes while minimizing adverse effects. A multicenter blinded RCT titled the COVID STEROID 2 trial was conducted to compare the required dosages of dexamethasone (36). This is the largest study to date that investigated the use of different dosages of corticosteroids in COVID-19 patients (36). It included 982 participants in an approximate 1:1 ratio (6 mg vs. 12 mg for up to 10 days, low vs. high dose respectively) (36). The outcomes included days alive without life support, such as MV or circulatory support, at 28 days (primary) and for up to 90 days (secondary) (36). In patients that are infected with COVID-19 and simultaneously hypoxemic (requiring >10 L of oxygen as part of their inclusion criteria) the high dose course of dexamethasone did not improve the primary outcome compared to the low dose course (22 vs. 20 days, high vs. low dose groups, respectively, with 1.3 days difference, 95% Cl: 0.0–2.6; *p* = 0.07) (36). An open label trial from the European Respiratory Journal with a similar design (200 participants, in an approximate 1:1 ratio) found that a high dose course (20 mg dexamethasone for 5 days followed by 10 mg for an additional 5 days) improved reduced clinical worsening when compared to a low dose course (6 mg once daily for 10 days), but not mortality at 28 days (84). In the high dose group, 16 out of 98 participants had clinically worsened versus 32 out of 102 participants in the low dose group (rate ratio, 0.427; 95% CI: 0.216–0.842; *p* = 0.014) (84). Another steroid agent of interest, methylprednisolone, was investigated in a trial that assigned patients to either pulsed IV methylprednisolone treatment, 250 mg/day for 3 days plus standard care, or standard care alone (82). Similar to the RECOVERY trial, they found that among those who received methylprednisolone, a higher percentage of patients improved (94.1% versus 57.1%), and a lower percentage of these patients suffered mortality (5.9% versus 42.9%; *p* < 0.001) (27 patients). These patients were recruited from a hospitalized population that were in the early pulmonary phase of the infection, meaning that this is before they required ventilation with or without intubation (82). It is important to mention however, that this study is limited by its small sample size of 68 (82).

Following the release of the RECOVERY trial, dexamethasone became the standard of care as most of the subsequent trials released analyzed the use of dexamethasone (85, 86). A few studies comparing dexamethasone to methylprednisolone were done. One trial named the MEDEAS trial corroborated the current guidelines suggesting the administration of agents equivalent to 6 mg of dexamethasone, by showing that higher dose methylprednisolone was not superior to low dose dexamethasone in improving outcomes (87). In another ambispective cohort study, 105 patients received high dose (250–500 mg) methylprednisolone for 3 days followed by 50 mg of oral prednisolone for 14 days (86). This was compared to another group of 111 patients who received 6 mg of dexamethasone 4 times daily for 7–10 days (86). The study included participants above the age of 18 who were hospitalized due to a PCR-confirmed COVID-19 pneumonia, supplementary oxygen requirements, and computed tomography (CT) confirmation of an imaging pattern of COVID-19 pneumonia (86). It was found that in both treatment groups, there was a decreased level of severity markers after 4 days of treatment that included: C-reactive protein (CRP), D-dimer and lactate dehydrogenase (LDH) (86). It was also found that the group that received methylprednisolone had decreased transfer to the ICU (4.8% vs. 14.4%), mortality (9.5% vs. 17.1%), and recovery time (3–4 days vs. 5–8 days *p* < 0.0001). Another study included 262 patients in 3 groups, with the participants being patients who were suffering from respiratory failure and had to be admitted to the intensive care unit (ICU) (85). The 3 groups included 104 receiving at least 1 mg/kg per day of methylprednisolone, 83 receiving at least 6 mg of dexamethasone for a duration greater than 7 days, and 75 patients in the usual care (control) group (85). The all-cause mortality at 50 days was found to be less in both corticosteroid groups when compared to usual care, and in patients that were mechanically ventilated, mortality was 42% lower in the methylprednisolone group (hazard ratio 0.48, 95% CI: 0.235–0.956, *p* = 0.0385) (85). These studies, while suggesting that methylprednisolone could hold superior value, are observational studies and lack the large, blinded, and experimental study design to provide strong evidence for superiority. The CAPE COVID trial was a French trial examining hydrocortisone in critically ill patients with COVID-19, and in contrast to a lot of the COVID-19 steroid trials it was double-blinded (88). The primary outcome, death or dependency on mechanical ventilation at day 21 (termed treatment failure) occurred less in the hydrocortisone group than in the control group (32 out of 76 versus 37 out of 73, respectively). It is important to note that this trial was undermined due to choices regarding terminate enrollment pending/following the expected release of data from the RECOVERY trial in June 2020 (88).

Most studies examining corticosteroids have used the systemic form in moderate-to-severe disease. The importance of the following studies is to highlight the role of inhaled corticosteroid agents in mild-moderate disease, specifically in the outpatient setting. In two studies, the STOIC trial and the PRINCIPLE trial, a proposed dosage of budesonide was 800 mcg twice daily (83, 89). The STOIC trial, a phase 2 open-label trial, included 146 participants with a mean age of 45 that were randomized in a 1:1 ratio to receive budesonide revealed that among patients with early COVID-19 disease, those treated with inhaled budesonide required less medical evaluation at 28 days, and only 1% required medical evaluation, versus 14% of patients who received usual care suggesting that inhaled budesonide may reduce the need for escalation of care (ED assessment or hospital admission) in mild-moderate disease (89). The PRINCIPLE trial recruited participants who were of an older age category in comparison to the prior study (PRINCIPLE inclusion criteria age greater than 65 or 50 with comorbidities vs. 18 in the STOIC trial) with a higher prevalence of comorbidities (mean age 64.2, 81% having comorbidities vs. less than 10% in the STOIC trial) (83, 89). There were 787 participants in the usual care group versus 1,069 in the budesonide group (83). A lower percentage of participants in budesonide arm (6.8%) versus the usual care arm (8.8%) (OR: 0.75 CI: 95%, 0.55–1.03) (83). The median time to reported recovery was also lower in the budesonide arm (11.8 days) compared to the in usual care arm (14.7 days) (HR 1.21; 95% CI: 1.08–1.36) (83). The conclusion is that in non-hospitalized patients inhaled budesonide reduces time to self-reported recovery, but has no clear effect on mortality or hospitalization (83). Both budesonide studies, similar to the smaller trials of systemic corticosteroids, are limited by their open label design, as well as small sample sizes (83, 89). This limitation is further exacerbated by the fact that the symptom alleviation was self-reported, making the clinical significance unclear in both of the aforementioned studies (83, 89). Furthermore, compared to the PRINCIPLE trial, the STOIC trial had participants that are relatively younger and healthier (less comorbidities) (83, 89). This may confound a lower number of events such as hospitalization or emergency department visits (83, 89).

### 6.2. Cons

Other studies failed to identify significant advantage of corticosteroids in COVID-19, as shown in Table 2. A noticeable trend of potential harm was observed in a recent cohort study of 19,973 patients with COVID-19 disease (90). It was found that patients who are not on oxygen therapy who received dexamethasone in the first 48 h had a 90 days mortality hazard ratio of 1.76 [95% CI (1.47–2.12)] in comparison to those who did not receive dexamethasone (90). Another trial from Argentina analyzed 98 participants who were assigned to either receive 16 mg of dexamethasone for 5 days, followed by 8 mg for 5 days, or 6 mg for 10 days (93). At 28 days after randomization, they found no difference between the 2 groups on ventilator free days, the primary outcome, mean duration of mechanical ventilation or all-cause mortality (93). It is important however to note that the trial was prematurely terminated due to a low enrollment rate of participants (93, 94). Several other studies emphasized that a prolonged higher dose of corticosteroids is less likely to be superior to conventional/low dose corticosteroid therapy, and in some cases might even be more harmful (87, 95, 96). One such study was a multicenter, open-label RCT which assigned patients to receive either a continuous infusion of 80 mg of methylprednisolone for a total of 8 days which then will be slowly tapered down, or 6 mg of dexamethasone once daily for a maximum of 10 days (87). 28 days mortality was similar in both groups, 10.4% versus 12.1%, in the methylprednisolone versus the dexamethasone groups, respectively, implying that a higher dose of corticosteroids does not necessitate better mortality results (87). Another study aimed to compare the same agent, dexamethasone, given at a high 20 mg dose versus low 6 mg dose, once daily (95). The 28 days mortality was again similar in the two groups, and the low dose group showed better outcomes in patients requiring oxygen supplementation (high-flow oxygen or noninvasive ventilatory support) compared to the high dose group (95). Furthermore, a multicenter placebo controlled RCT (COVIDICUS) showed that high dose dexamethasone possesses no increased benefit in improving 60-day survival rate [HR = 0.96, 95% CI (0.69–1.33); *p* = 0.79] for patients in the intensive care unit suffering from acute hypoxic respiratory failure induced by a severe COVID-19 infection (96). As for the hospitalized patients admitted for COVID-19 induced hypoxia, not requiring ventilation (no oxygen or simple oxygen support), the RECOVERY collaborative group showed that using a higher dose of corticosteroids increases the risk of death as compared to low dose corticosteroids (97). The study was a controlled, randomized and open-label trial which recruited a total of 1,272 hypoxic patients with COVID-19, requiring simple oxygen or no oxygen support, to either receive standard care, plus 20 mg of dexamethasone once daily for 5 days then 10 mg for 5 days or until discharge (*N* = 659, high dose group), or 6 mg of dexamethasone once daily for 10 days or until discharge (*N* = 613, low dose group) (97). One hundred and twenty three patients in the high dose group versus 75 patients in the low dose group died [RR = 1.59, 95% CI (1.20–2.10)]; *p* = 0.0012 (97). Additionally, higher percentage of non-COVID related pneumonia was reported, 10% versus 6%, in the high dose versus low dose group, respectively. Also, the risk for hyperglycemia was significantly higher for the high dose group, 22% vs. 14% in the low dose group (97). other literature reviews asserted the dangers of prolonged high dose corticosteroid therapy in the setting of COVID-19, among the aforementioned risks for developing pneumonia and hyperglycemia, other adverse side effects include, but are not limited to myocardial infarction, bacterial sepsis, cerebrovascular and ophthalmic complications among others (97–99). Additionally, one of the agents mentioned prior, hydrocortisone, was studied in the REMAP-CAP trial, whereby it has shown no benefit (100). The aforementioned trial is an open label trial that randomized 403 patients to one of three treatment arms: either a fixed-dose course, a shock dependent course, or a third group receiving no hydrocortisone (100). There were no differences between the three arms in their primary or secondary outcomes, which were median number of organ support-free days and in-hospital mortality (100). Furthermore, in one double blind randomized control (RCT) trial, inhaled ciclesonide was used at a dosage of 320 mcg (160 mcg per actuation) twice daily for 30 days (*N* = 197) (91). When compared to the use of a placebo metered dose inhaler (MDI), the median self-reported time to recover from all COVID-19 related symptoms was 19 days in both groups (91). Another RCT titled “CONTAIN” randomized 203 participants to either 600 mcg of inhaled ciclesonide and 200 mcg intranasally, or to a placebo MDI and nasal saline for a duration of 14 days (101). They did not find a significant difference in symptom resolution at day 7 between the two groups (40% vs. 35%, treatment group vs. placebo group, respectively) (101). The prior results were further reinstated in a more recent multicenter, randomized, controlled and open-label trial which studied the duration of oxygen supplementation as the primary outcome and death/requirement of mechanical ventilation as the secondary outcome following administration of 340 mcg of inhaled ciclesonide, twice daily, for 14 days versus standard treatment alone (102). The reduction in the duration of oxygen supplementation was less than 1 day for the ciclesonide group (*N* = 48) versus the standard treatment group (*N* = 50) [95% CI (0.47–1.11)], and 3 patients received ventilatory support/died equally in both groups [95% CI (0.15–5.32)] (102). As for patients presenting with mild/asymptomatic COVID-19, administering 400 mcg of ciclesonide for 7 days has lead to poorer imaging results of pneumonia compared to the control group (94). Exacerbated pneumonia was observed in 39% of the patients in the ciclesonide group compared to 18.8% in the control group [RR = 2.08, 95% CI (1.15–3.75)] (94). Consequently, not only inhaled ciclesonide was proven to be ineffective, but it has also worsened the signs of pneumonia in mild/asymptomatic cases (94, 102). Also, another decentralized placebo-controlled RCT aimed to explore inhaled fluticasone therapy, on an outpatient level, in the setting of mild/moderate COVID-19 infection, specifically during the omicron and delta strains (103). Patients were divided among two groups, placebo (*N* = 621) or inhaled fluticasone therapy (*N* = 656), and the time to recovery was similar among the two groups [HR = 0.56, 95% CI (0.91, 1.12)] (103). Additionally, single-blind RCT was conducted in China utilizing methylprednisolone as the corticosteroid treatment of choice for COVID-19 patients with CT scan confirmed pneumonia (104). In the latter study 43 patients were assigned to receive 1 mg/kg per day of IV methylprednisolone, while another 43 patients received no treatment (saline) (104). Results showed that there was no difference in the primary outcome, clinical deterioration at 14 days, among the two groups (4.8% vs. 4.8%, OR 1.0; 95% CI, 0.134–7.442; *p* = 1.00) (104). The difference in secondary outcomes such as ICU admission, mortality in-hospital, hospitalization stay among others, was also insignificant (104). Consequently, the prior study showed that giving methylprednisolone will not improve the clinical deterioration at 14 days in COVID-19 patients, yet its credibility is limited by the low sample size and premature termination due to the decline in COVID-19 cases (104). Another single-blind RCT was conducted in Iran on moderate-to-severe COVID-19 cases (92). The patients were randomly assigned to one of the three treatment regimens: 8 mg of dexamethasone once (low dose group, *N* = 47), twice (intermediate dose group, *N* = 40) or thrice (high dose group, *N* = 46), daily, IV, for 10 days utmost or until discharge (92). The low dose group showed better results in terms of the primary and secondary outcomes, clinical response (HR = 2.03, 95% CI: 1.23–3.33, *p* = 0.03) and mortality (HR = 0.36, 95% CI: 0.15–0.83, *p* = 0.02), respectively (92). The aforementioned study displays that high dose dexamethasone has the protentional to increase drug-associated side effects as well as reduce survival rate, but the study remains limited by the recruitment of a very low number of patients (92). Lastly, some articles declared that using steroids such as dexamethasone during the early stages of a COVID-19 viremia may extend the replication period of the virus due to the suppression of the immune response, thus leading to more severe outcomes (104–108). Therefore, more importance should be placed on deciding the time of initiation of corticosteroid therapy, as well the dosages and routes of administration, because the aforementioned parameters highly dictate the progression of the disease, as well as the severity of the adverse events (104–108).

**Table 2:** High impact studies that failed to show positive impact of steroids in COVID-19.

Study	Study type	Country	Study size	Agent used	Primary outcome
Dexamethasone in hospitalised COVID-19 patients not on intensive respiratory support (90)	Cohort study	United States	19,973	Dexamethasone administered in the first 48 h following hospitalization	Increased 90-day mortality risk for patients on no oxygen
Efficacy of inhaled ciclesonide for outpatient treatment of adolescents and adults with symptomatic COVID-19: a randomized clinical trial (91)	Multicenter, double-blind RCT	United States	400	A daily dose of 640 mcg of inhaled ciclesonide (160 mcg/actuation, 2 actuations twice daily)	No difference in recovery time from COVID-19 related symptoms between ciclesonide and placebo group
Comparing efficacy and safety of different doses of dexamethasone in the treatment of COVID-19: a three-arm randomized clinical trial (92)	RCT	Iran	144	Dexamethasone (8 mg) once, twice or thrice	Worse survival rates and greater adverse reactions encountered when giving higher dosages of dexamethasone

Furthermore, it is important to highlight the possible side effects of steroids. For COVID-19 patients, in particular, giving a corticosteroid such as dexamethasone was associated to an increased risk of acquiring an invasive fungal infection and superinfection (109). One multicenter, observational, retrospective study examined the incidence of superinfections in COVID-19 patients (*N* = 155) above the age of 18, admitted to the hospital requiring mechanical ventilation (109). Superinfections were detected in 61% (44/72) versus 28% (23/83) of patients (*p* < 0.0001), in the dexamethasone versus no dexamethasone arms, respectively (109). Additionally, invasive fungal infections were detected exclusively in the group of patients who received dexamethasone, 11% (8/72) versus 0% (0/83) (*p* < 0.0001), in the dexamethasone versus no dexamethasone arms, respectively (109). Furthermore, several case reports aimed at exploring the risk of developing mucormycotic and aspergillosis in COVID-19 patients following the administration of steroids. One such report described a dual fungal infection (mucormycosis and aspergillosis) alongside a superimposed klebsiella infection in a 67 years-old diabetic woman with moderately severe COVID-19 infection (110). Early detection and management with antifungal agents such as voriconazole and amphotericin B was proven to be effective for treating the aforementioned case (110). Another case report described the development of maxillo-fascial mucormycosis in a 54 years-old male patient who was hospitalized following severe COVID-19 pneumonia (111). The fungal infection was likely due to the patient’s long-term intake of high dose systemic steroids (111). A subsequent literature search on the development of mucormycosis in 100 COVID-19 patients in India (taken from 30 publications), identified the use of corticosteroids as the biggest risk factor, since it was observed in 90.5% of the reported patients (111). Another review article further emphasized the association between corticosteroid usage in COVID-19 patients and subsequent mucormycosis infection which stated that the latter is linked to higher fatality rate and further deterioration of the pandemic (112). All the aforementioned data warrants further investigation into the best possible treatment regimen for patients who are at risk of developing an invasive fungal infection such as diabetics, and for those who corticosteroids are not an option.

## 7. Conclusion

Despite the outstanding universal effort put in favor of minimizing the morbidity and mortality caused by COVID-19 through global vaccination campaigns, yet the search for an effective therapeutic agent remains on the rise. Although many trials have demonstrated a statistically significant result for their primary outcome (decrease in mortality or ventilatory-free days), the benefit of such studies remains questionable due to low number of participants, the open-label study design and early termination following the RECOVERY trial results, or due to a decline in COVID-19 cases. Furthermore, myriad studies showed mixed results, with some reaching secondary, not primary outcomes, and vice versa. Plus, the beneficial dosages remain under suspicion with some trials showing greater risk for higher dosages of corticosteroids. Hence, our review of the literature has concluded that there might be a potential benefit for the use of systemic corticosteroids in the context of a severe COVID-19 pneumonia. However, it is important to push for high quality multi-center placebo-controlled randomized clinical trials to better understand the effectiveness of corticosteroids in the setting of COVID-19.

## Author contributions

MA developed the idea and reviewed the framework. AB and YF wrote the first draft of the manuscript. RZ created and edited the figures. RZ, FB, and MA did the final editing. AB, YF, RZ, FB, and MA contributed to corrections and adjustment of subsequent iterations of the manuscript. All authors contributed to the article and approved the submitted version.

## Conflict of interest

The authors declare that the research was conducted in the absence of any commercial or financial relationships that could be construed as a potential conflict of interest.

## Publisher’s note

All claims expressed in this article are solely those of the authors and do not necessarily represent those of their affiliated organizations, or those of the publisher, the editors and the reviewers. Any product that may be evaluated in this article, or claim that may be made by its manufacturer, is not guaranteed or endorsed by the publisher.
